# Characterization of the Myometrial Transcriptome of Long Non-coding RNA Genes in Human Labor by High-Throughput RNA-seq

**DOI:** 10.1007/s43032-022-00910-5

**Published:** 2022-04-25

**Authors:** Yihong Luo, Long Cui, Lina Chen, Lele Wang, Kaiyuan Ji, Huishu Liu

**Affiliations:** 1 Guangzhou Key Laboratory of Maternal-Fetal Medicine, Guangzhou Women and Children’s Medical Center, Guangzhou Medical University, Guangzhou, China; 2 School of Medicine, South China University of Technology, Guangzhou, China

**Keywords:** Parturition, lncRNA, RNA sequencing, Myometrium

## Abstract

The contraction of myometrium is pivotal in expelling the fetus and placenta during labor, but the specific mechanism of myometrium changing from quiescent to a contractile state is still unclear. Previous studies have shown that changes in certain genes or proteins are related to the regulation of myometrial contraction, which are considered to be contraction-associated genes. Long non-coding RNAs (lncRNAs) are increasingly recognized as important molecular players in regulating gene expression and many biological processes, but their roles in the rhythmic contraction of myometrial cells during labor remain to be explored. This study aimed to reveal the differentially expressed lncRNAs in the human myometrium of non-labor (NL, *n* = 9) and in-labor (IL, *n* = 9). Furthermore, bioinformatic analysis of lncRNA targeted mRNAs was performed to explore the biological processes and pathway alterations during labor. The results showed a total of 112 significantly differentially expressed lncRNAs between two groups were identified, of which 69 were upregulated and 43 were downregulated in IL group, compared with NL group. In addition, the enrichment analysis of Gene Ontology (GO) and pathways showed that the lncRNAs corresponding targeted mRNAs were associated with mRNA splicing, splicesome, ferroptosis, FGFR and NOTCH signaling pathways. Our study constitutes the first report on investigating the gene expression landscape and regulatory mechanism of lncRNAs within laboring and non-laboring myometrium using RNA sequencing (RNA-seq) and bioinformatic analysis. This study provided high-throughput information on the lncRNA in the myometrium of women in labor and those not in labor, to discover novel lncRNA candidates and potential biological pathways involved in human parturition.

**Supplementary Information:**

The online version contains supplementary material available at 10.1007/s43032-022-00910-5.

## Introduction

Parturition is a complex process involving myometrial activation, cervical ripening, and membrane/decidual activation. The transition of the myometrium from quiescence to the highly contractile labor state is thought to be controlled at the transcriptional level through changes in the expression of specific genes whose products increase contractibility and excitability. High-throughput RNA sequencing (RNA-seq) can provide an opportunity to examine the gene expression landscape within laboring and non-laboring myometrium to identify the gene sets controlling labor. Transcriptional differences between laboring and non-laboring human myometrium have been examined by multiple studies before [1–3]. On aggregate, studies have shown that the labor is associated with inflammatory signals, including genes/pathways related to cytokine signaling, chemotaxis, and immune response. The maintenance of myometrial quiescence and the timing of labor involve a delicate balance between hormonal, inflammatory, and physical factors that regulate integrated signaling pathways between the mother and the fetus [4, 5].

Long non-coding RNAs (lncRNAs) are a type of RNAs that transcript longer than 200 nt nucleotides without translated proteins. The expression of lncRNAs is spatiotemporally and tissue-specific and plays important roles in many aspects, including epigenetic regulation, transcriptional regulation, and post-transcriptional regulation [6]. With the rapid development of next-generation sequencing technologies, lncRNAs have been examined as novel regulatory players in cellular and biological processes and have plenty of functions in various physiological and pathological processes [7–10]. These evolutionarily conserved regulators of gene expression play important parts in a variety of biological and pathological processes, including cell differentiation, cancer, immune regulation, and female reproduction [11–13].

Previous studies have utilized the Illumina® expression microarray platform to identify differential expression of coding and non-coding RNAs between the myometrium of women not in labor and those in labor (fold change > 1.25, *q*-value < 0.1); a total of 13 differentially expressed lncRNAs were identified, two of which contained co-differential expression at genomic loci that contain coding-non-coding gene pairs [14]. Limited to microarray technology, lncRNA found is limited even in the low standard of difference. William E Ackerman et al. [15] were profiled RNAs (including mRNA, miRNA, and lncRNA) in myometrial biopsies collected from women spontaneous term labor, term non-labor, and spontaneous preterm birth using RNA sequencing, but only mRNA and miRNA were analyzed, while lncRNA was neglected. The biological processes, molecular functions, and pathways associated with term parturition of lncRNAs have not been described, and little is known about its regulatory mechanisms. We undertook this study in order to explore the potential role of lncRNAs and their regulatory mechanisms during pregnancy and labor.

## Materials and Methods

### Study Design and Objectives

A total of 18 participants were recruited, and they had cesarean section in Guangzhou Women and Children’s Medical Center (Guangzhou, China) for reasons of breech, placenta previa, fetal distress, or cephalopelvic disproportion. All the parturients were full-term (≥ 37 weeks), singleton, primipara, no cesarean section or any uterine surgery before, no clinical and histological chorioamnionitis or any medical complication (including gestational diabetes, preeclampsia, and thyroid disorder), and delivered appropriate for gestational age neonates (AGA, defined as a birthweight between the 10th and 90th percentile for the gestational age at birth [16]). The participants were categorized into two groups according to their labor stages: in-labor (IL, *n* = 9) and non-labor (NL, *n* = 9). Labor was defined by the presence of regular uterine contractions associated with progressive cervical dilation, and cervical change due to contractions. In addition, the criterion of cervical dilatation ≥ 2 cm was used to rule out the possibility of false labor. Non-labor women presented with no palpable uterine contractions and a closed cervix.

Myometrial tissue samples were collected in the lower uterine segment during cesarean section following delivery of the placenta from the midpoint of the superior aspect of the uterine incision and measured approximately 1.0 cm^3^. Tissues were immediately washed with phosphate-buffered saline buffer (Sigma) to minimize blood and decidua content, then immersed in RNAlater (Sigma) and kept at − 80 °C until use.

All women provided written informed consent prior to the collection of myometrial samples. The collection and utilization of the samples for research purposes was approved by the ethics committee of Guangzhou Women and Children medical center (No. 2018041701).

### Total RNA Extraction and Long Non-coding RNA Library Construction

Total RNA was extracted from the tissues using Trizol (Invitrogen, Carlsbad, CA, USA) according to the manual instructions. For tissue samples, grind about 60 mg with liquid nitrogen into powder; then, centrifuge with 1.5 mL Trizol reagent at 12,000 × g for 5 min at 4 °C. The supernatant was added 0.3 mL of chloroform/isoamyl alcohol (24:1) and centrifuged at 12,000 × g for 10 min at 4 °C; the aqueous phase was transferred to a new tube and added equal volume of supernatant of isopropyl alcohol before being centrifuged at 12,000 × g for 20 min at 4 °C, and then the supernatant was removed. After washing with 1 mL 75% ethanol, the RNA pellet was air-dried and dissolved by adding 25 ~ 100 µL of DEPC-treated water. Subsequently, total RNA was qualified and quantified using a Nano Drop and Agilent 2100 bioanalyzer (Thermo Fisher Scientific, MA, USA).

According to the manufacturer’s instructions, ribosomal RNA (rRNA) was removed by using target-specific oligos and RNase H reagents to deplete both cytoplasmic (5S rRNA, 5.8S rRNA, 18S rRNA, and 28S rRNA) and mitochondrial ribosomal RNA (12S rRNA and 16S rRNA) from total RNA preparations. Following SPRI bead purification, the RNA is fragmented into small pieces using divalent cations under elevated temperature. The cleaved RNA fragments are copied into first strand cDNA using reverse transcriptase and random primers, followed by second strand cDNA synthesis using DNA Polymerase I and RNase H. This process removes the RNA template and synthesizes a replacement strand, incorporating dUTP in place of dTTP to generate ds cDNA. These cDNA fragments then have the addition of a single 'A' base and subsequent ligation of the adapter. After UDG treatment, the incorporation of dUTP quenches the second strand during amplification. The products are enriched with PCR to create the final cDNA library. The libraries were assessed of quality and quantity in two methods: check the distribution of the fragment size using the Agilent 2100 bioanalyzer, and quantify the library using real-time quantitative PCR (QPCR) (TaqMan Probe). The qualified libraries were sequenced pair end on the BGISEQ-500/ MGISEQ-2000 System (BGI-Shenzhen, China).

### RNA-Sequencing Data Analysis

The results of raw data sequences were carried out through transcriptomic analysis using the bioinformatics approach through Linux, R and windows operating systems. FastQC was used to perform quality control assessments on raw sequence data. HISAT2 based on the H. sapiens UCSC hg38 reference (GRCh38) genome (https://ccb.jhu.edu/software/hisat2/index.shtml) was used for the process of gene mapping alignment. Alignment calculations of non-coding RNA were performed using featureCount based on Ensembl genome browser 84 in GTF file format (ftp://ftp.ensembl.org/pub/release-84/gtf/homo_sapiens). The raw data is available in GEO repositories; the accession number is GSE181348, (https://www.ncbi.nlm.nih.gov/geo/query/acc.cgi?acc=GSE181348). The lncRNA quantitative expression values were calculated for each sample based on fragments per kilobase of exon per million fragments mapped (FPKM) method [17]. The differentially expressed lncRNAs in IL vs NL groups were identified using two-sided Student’s *t*-test of OmicShare tools (http://www.omicshare.com/tools), based on two criteria of twofold change (FC) increase or decrease in expression levels, and *p* < 0.05. The lncRNAs corresponding targeted mRNAs were obtained from NPInter database [18]. The lncRNA-mRNA interaction network was formed by Cytoscape software (version 3.7.2) [19]. Gene Ontology enrichment analysis was carried out using DAVID tools [20]. The KEGG pathway enrichment analysis was performed using KOBAS (3.0) [21]; adjusted *p* < 0.05 was considered significant.

### Quantitative Real-Time PCR (qRT-PCR)

Total RNA was extracted from tissues with ES-RN002plus (YISHAN) according to the manufacturer’s instructions. One microgram of total RNA was reversely transcribed into cDNA and subsequently analyzed by qRT-PCR. The qRT-PCR was performed on a ABI QuantStudio6 with TB Green™ Premix Ex Taq™ II (Tli RNaseH Plus) (TaKaRa). All the primers used in the study are available in the Table 1. PCR conditions included denaturing at 95 °C for 1 min, and 40 cycles of denaturing at 95 °C for 15 s followed by annealing and extension at 60 °C for 30 s. Relative gene expression was calculated using the Livak and Schmittgen 2^−ΔΔ^Ct method, normalized with the reference gene β-Actin.

**Table 1:** Sequences of primers used in PCR reactions

	Forward primer (5′-3′)	Reverse primer (5′-3′)
SNHG8	ATCCAAGTGGTAATGGGCGA	GAACACCCGTTTCCCCAACT
SNHG3	CTGTTTTGCAGAAAGTCTGCTG	ACCAACACAGTGTGCCTTCT
SNHG15	TGGCAGACCTGTACTCCGTA	GGTGGATGACTAGACTGCCG
PGM5-AS1	TGGTACTTTCAGCCTGTCCG	AACAGACGGCTTCAGTGGTT
LOC107985064	CCAGATGGCTGCAGGACTTT	ATTTCACTGGGCCCCAACTT
LOC105374235	TCACTCCTCTGCATTCACCAA	GCTCTGCAAAAATCCTCCTGTG
LINC01088	GCCTGGCTATCCTGGAGTTT	GGGCTTAGCTGTAAGGACGAA
LINC00595	CCAAGTGGGCTGTGAAGTGT	TTCTACATGGCTGTCACCCG
CLRN1-AS1	GTGTCACTTGGTAACAAAGGTCG	AAAGCCAACAACTGCCTCCT
ADAMTS9-AS1	GTTCCGATCTGACAGCCCAC	GAGCAGATTAGCTTTGCAGGG
β-actin	GGCCCAGAATGCAGTTCGCCTT	AATGGCACCCTGCTCACGCA

### Statistical Analysis

Group comparisons were performed using the *t* test for two groups by SPSS 20.0; data were expressed as means ± standard error; *p* < 0.05 was considered significant.

## Results

The clinical and demographic characteristics of each participant are displayed in Supplemental Table 1. Statistical analysis results showed there were no statistically significant differences in maternal age, parity, BMI, gestational age (GA) at delivery, and birth weight between the non-labor and in labor groups (*p* > 0.05) (Table 2).

**Table 2:** Clinical characteristics of the study participants

	Non-labor (*n* = 9)	In labor (*n* = 9)	*p* value
Maternal age (years)	29.56 ± 4.65	29.11 ± 2.60	0.8163
Parity	1.44 ± 0.68	1.56 ± 0.96	0.7927
BMI (kg/m^2^)	25.08 ± 1.86	25.60 ± 1.76	0.5763
Gestational age at delivery (weeks)	38.75 ± 0.43	38.89 ± 0.74	0.7152
Birth weight (g)	3215.56 ± 410.45	3012.22 ± 318.39	0.2846

Abbreviations: *BMI*, body mass index

Data is expressed as mean ± SEM

### Differentially Expressed lncRNAs

**Fig. 1:**
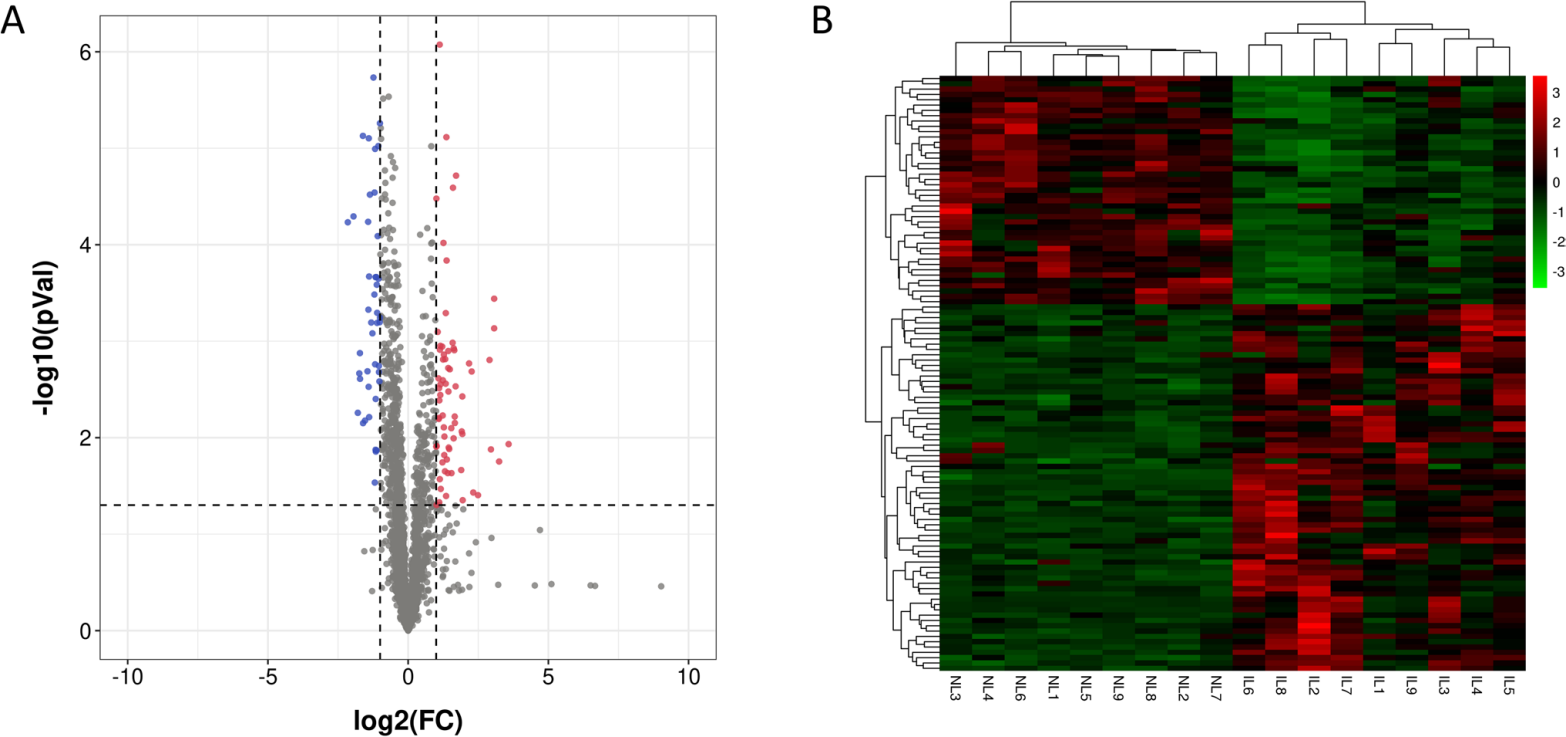
RNA sequencing analysis of the long non-coding RNA gene expression profiles of myometrium at term non-labor (NL) and in labor (IL). **A** Volcano plot showing the ratio between the average gene expression of NL and IL groups (*X*-axis) vs. the significant *p* values from the moderated *t*-test. **B** Heatmap of gene expression in NL and IL clustered by the long non-coding RNA genes. Rows correspond to genes while columns correspond to samples. High expression levels are shown in red, while low expression levels are in green

**Fig. 2:**
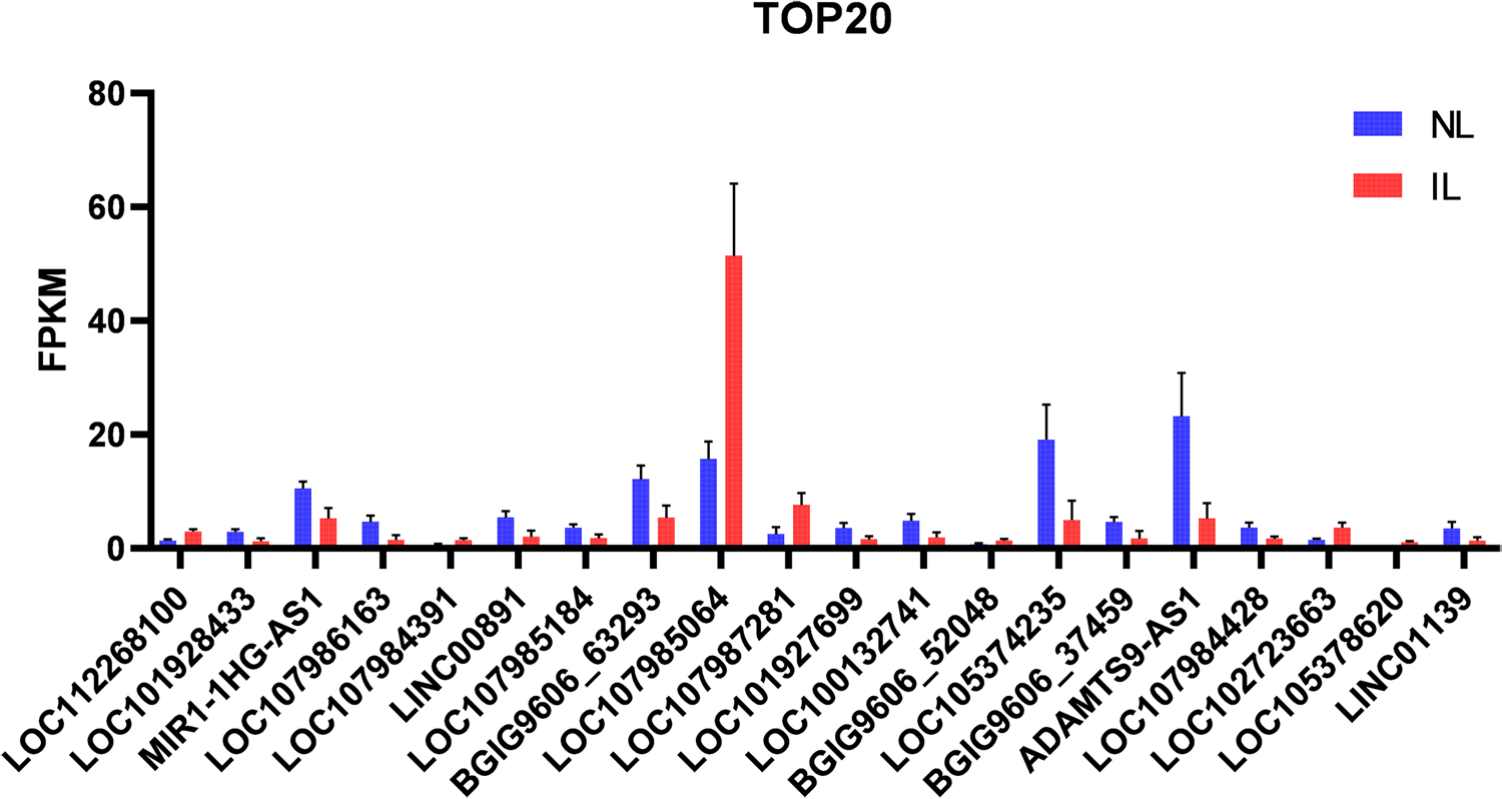
Top 20 differentially expressed lncRNA sort by *p* value of myometrium between NL and IL

To establish the differentially expressed lncRNAs of human myometrium between pregnant women with and without labor, RNA-seq on lncRNAs was performed. A total of 2750 distinct lncRNAs were detected after excluding lncRNAs that had extremely low abundances (average FPKM < 1 in both NL and IL) (Supplemental Table 2). The were 112 lncRNAs significantly differentially expressed between the NL and IL groups (*P* < 0.05, |fold change|> 2), of which 69 were upregulated and 43 were downregulated in IL group compared to NL group (Fig. 1A and B, Supplemental table 3). These differentially expressed lncRNAs might play a role in maintaining the quiescent state or regulating the contractile state of myometrium, especially those with lower *p* values. The top 20 differentially expressed lncRNAs with the lowest *p* values are shown in Fig. 2.

### Target Gene Prediction of Differentially Expressed lncRNAs and Functional Analysis

**Fig. 3:**
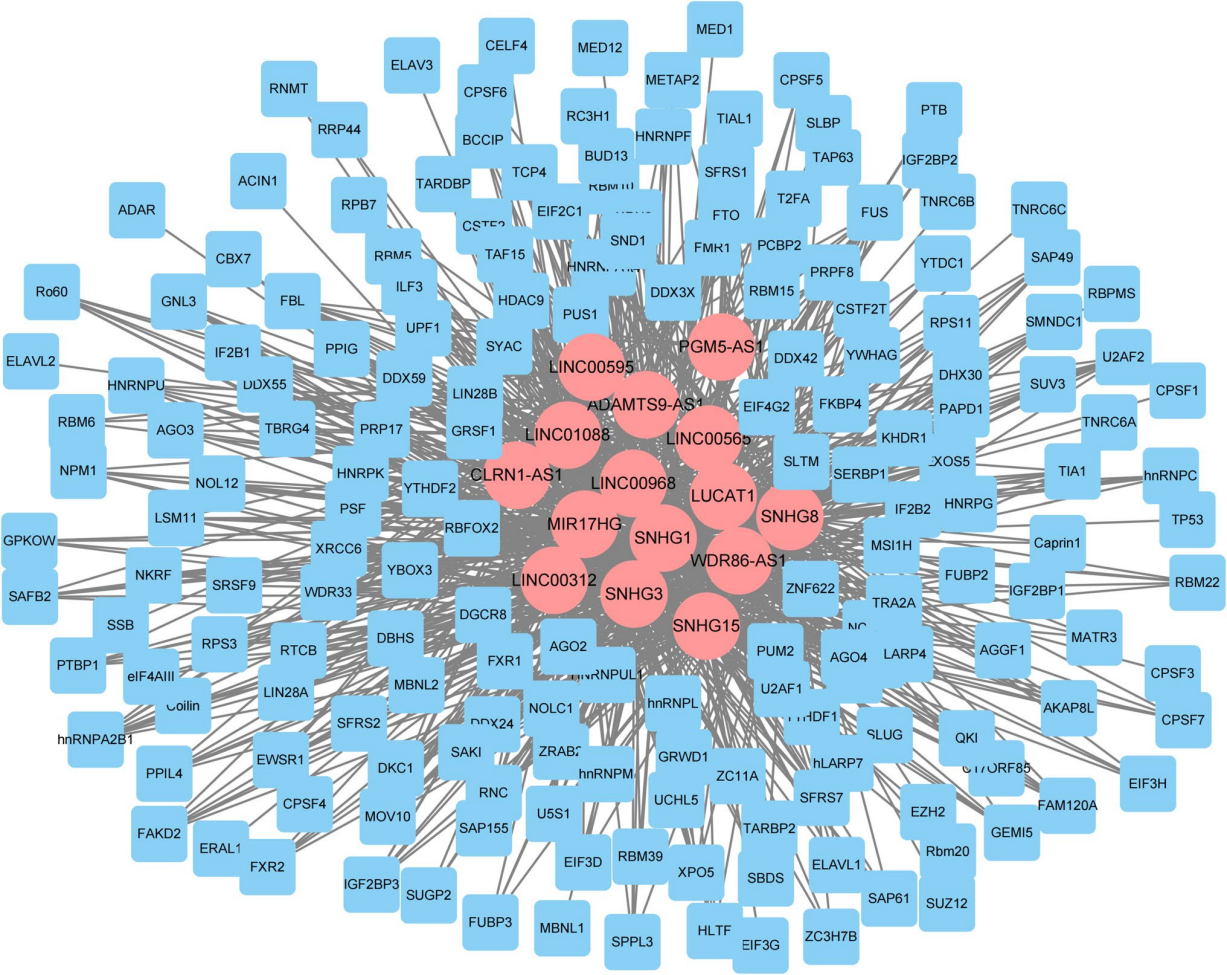
The lncRNA-mRNA pairs are shown in the network. Blue node represents mRNA; red node represents lncRNA

**Fig. 4:**
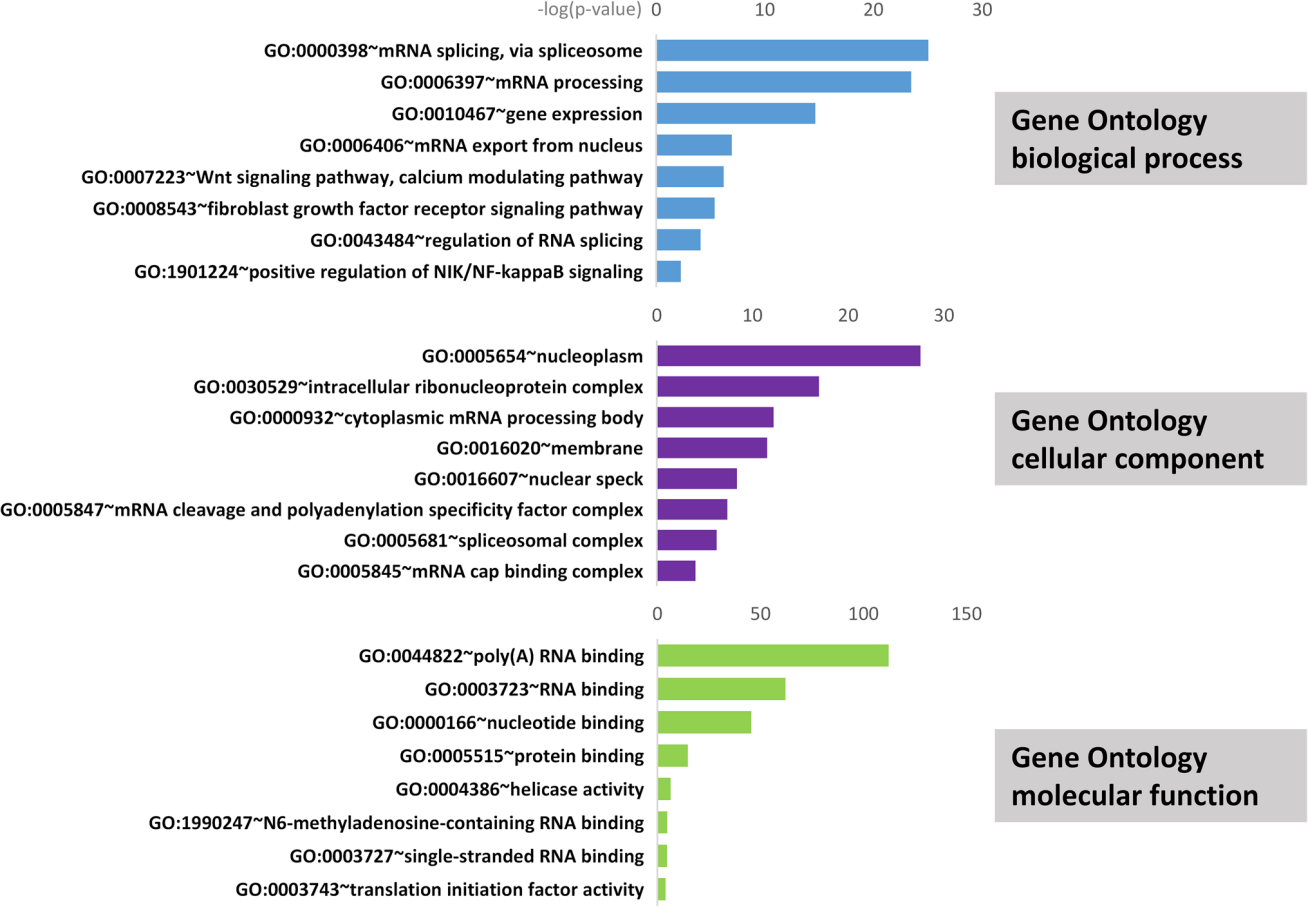
Gene Ontology (GO) analysis of differentially expressed lncRNAs corresponding target mRNAs in biological process (BP), cellular component (CC), molecular function (MF) categories

**Fig. 5:**
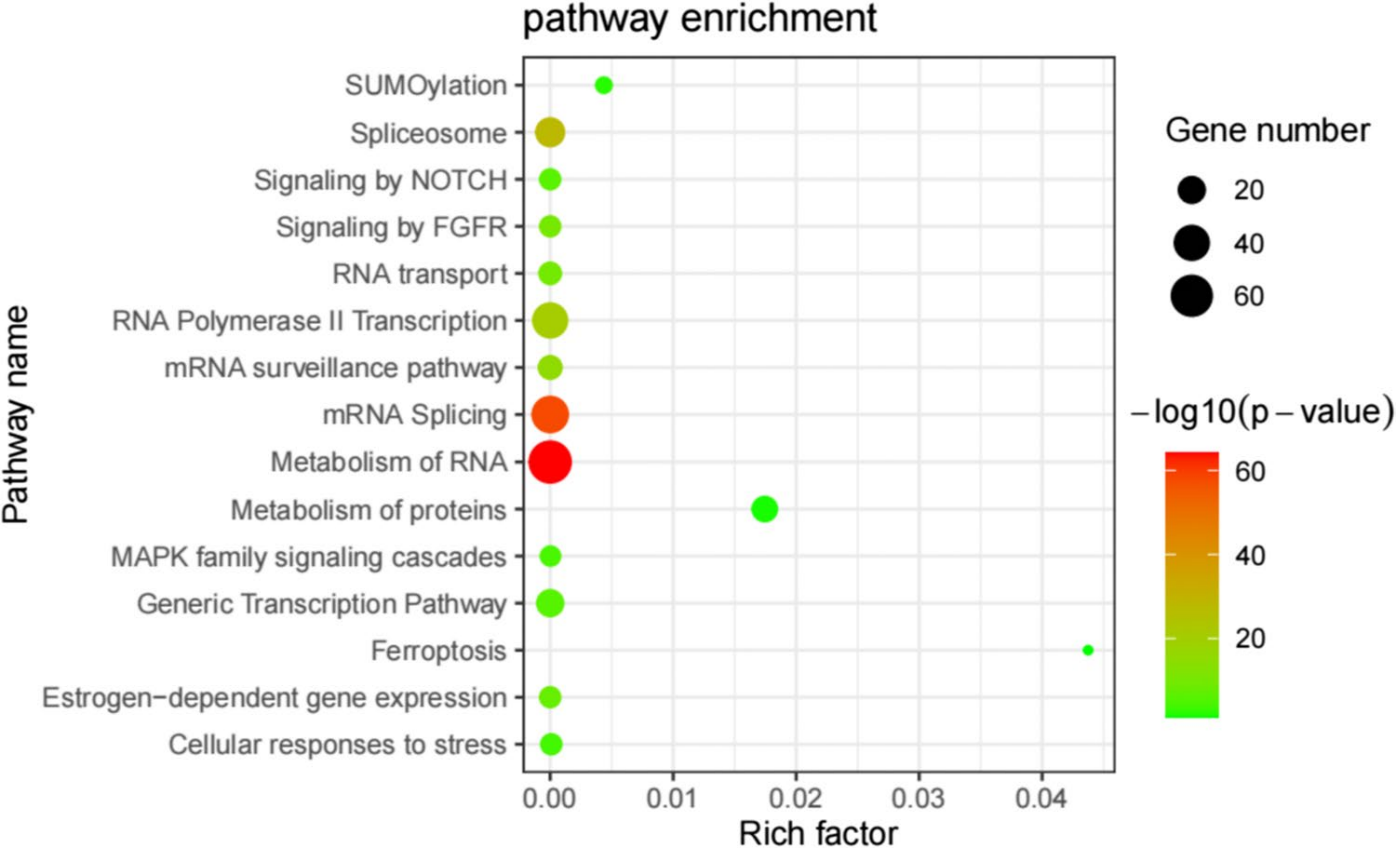
The signaling pathways of differentially expressed lncRNAs corresponding target mRNAs by KEGG and REACTOME pathway analysis

LncRNA can competitively bind to miRNA, thus effectively controlling the subsequent post-transcriptional regulation of miRNA and reducing the inhibition of miRNA on mRNA expression [22]. In order to gain further insight into the functional differences associated with lncRNAs during labor, NPInter database was used to predict the potential target mRNAs of these differentially expressed lncRNAs. A total of 15 lncRNAs and 185 target mRNAs were found to have interactions (Fig. 3, Supplemental table 4). Then, Gene Ontology enrichment analysis was carried out. Significantly enriched GO biological process terms of these target mRNAs mainly include regulation of mRNA splicing, Wnt signaling pathway, and NIK/NF-kappaB signaling. Cellular component was enriched in nucleoplasm, cytoplasmic mRNA processing body, and spliceosomal complex. Molecular function was enriched in RNA binding, helicase activity, and translation initiation factor activity (Fig. 4, Supplemental table 5). REACTOME and KEGG pathway analysis showed that the target mRNAs were enriched in SUMOylation, spliceosome, RNA transport, MAPK, FGFR, and NOTCH pathways (Fig. 5, Supplemental table 6).

### Verification of lncRNA Expression Profiles Using qRT-PCR

**Fig. 6:**
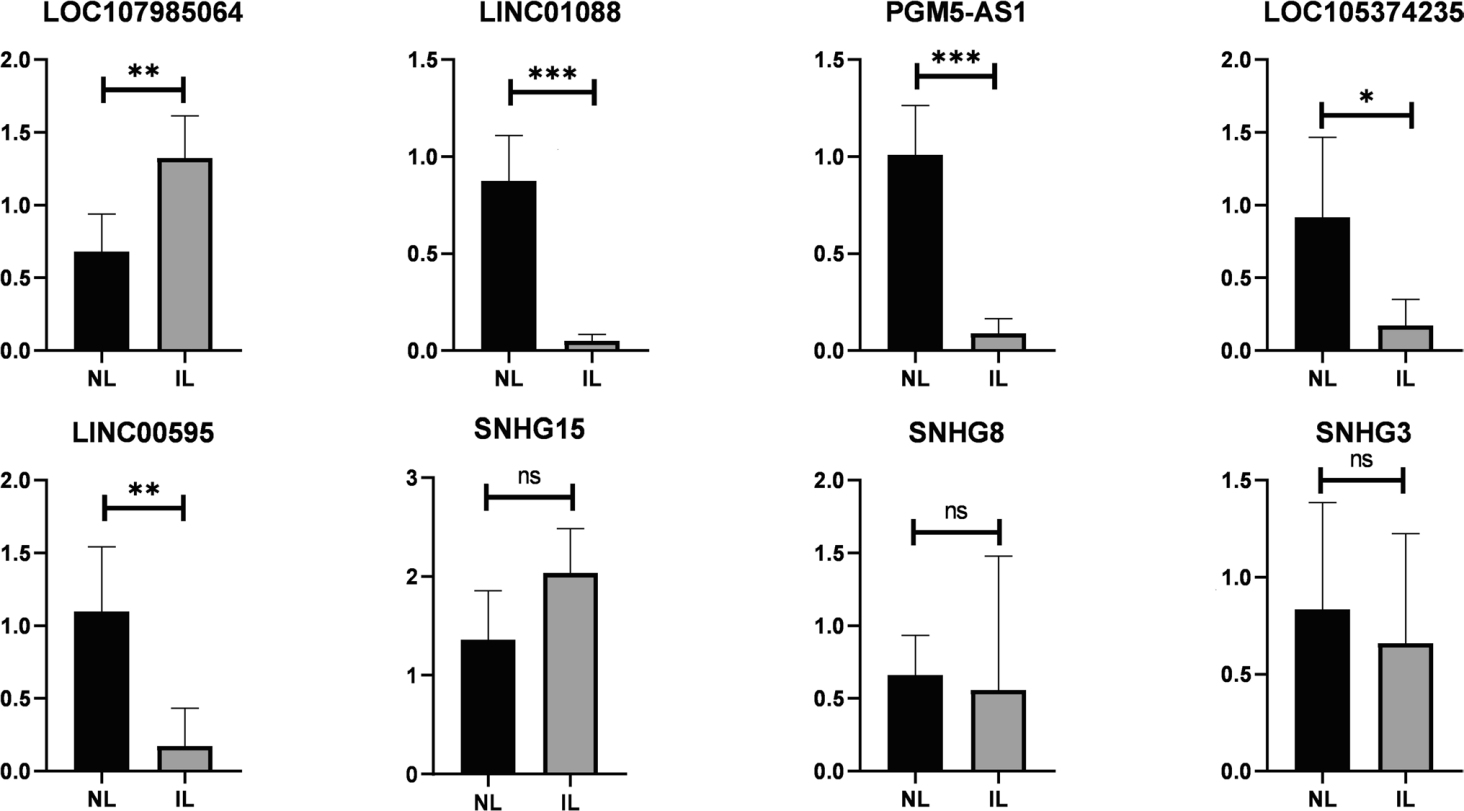
lncRNA expression levels of selected lncRNAs from the top 20 differentially expressed lncRNAs list and cores of lncRNA-mRNA interaction network were validated by RT-qPCR experiment by normalizing against β-Actin expression level (ns *P* > 0.05, * *P* < 0.05, ** *P* < 0.01, *** *P* < 0.001)

To verify the RNA-seq data, 10 lncRNAs (LOC107985064, LINC01088, PGM5-AS1, LOC105374235, LINC00595, SNHG15, SNHG8, SNHG3, CLRN1-AS1, and ADAMTS9-AS1) were selected from the top 20 differentially expressed lncRNAs list and cores of lncRNA-mRNA interaction network. Their relative expression levels were quantified by RT-qPCR in human myometrium between women non-labor (*n* = 5) and in labor (*n* = 5) (Fig. 6). Of the 5 selected differentially expressed lncRNAs (LOC107985064, LINC01088, PGM5-AS1, LOC105374235, and LINC00595), the expression levels exhibited the same trends of up- and downregulation as the RNA-seq data, which indicated a significant difference between NL and IL groups. The expression of CLRN1-AS1 and ADAMTS9-AS1 was too low to be detected.

## Discussion

In our study, we sequenced transcriptome of lncRNAs isolated from human myometrial samples taken from patients who were in labor or not. Activation of myometrium occurs at the onset of labor. In previous studies, microarray and RNA-seq were used to examine the changes in coding genes in myometrium during labor, in which there were obvious differences in the transcriptome once labor has been initiated [23, 24]. The maintenance of contraction in myometrium involved intricate gene regulatory networks. LncRNAs have novel regulatory roles in biological process and may serve as potential prognostic and therapeutic targets in clinical practice [25]. LncRNAs have also been sequenced in the myometrium of labor and non-labor myometrium using microarray or RAN sequencing techniques [14, 15], but their function has not been explored. Our results showed that the expression of 112 lncRNAs at term delivery was significantly different from that of women undergoing labor or not, counting 69 upregulated and 43 downregulated lncRNAs in detail. We performed a preliminary investigation of lncRNA expression in regulating physiological changes in labor and predicted their functions by analyzing their target mRNAs. From the results of enrichment pathways and GO terms of the target mRNAs of lncRNAs, we found mRNA splicing stands out well. mRNA splicing plays important roles in co-transcriptional and post-transcriptional regulation of gene expression, such as contraction of smooth muscle, and its role in regulating smooth muscle contraction has been reported [26–28]. In addition, myometrial cells can release multiple cytokines and chemokines, and provide a strong signal for activation of immune cells during labor [5]. NF-kappaB and NOTCH signaling pathways were significantly clustered in our analysis, which were well-known central signal pathways in the deployment of an inflammatory response [29–31]. The lncRNAs clustered at these function terms might be key regulators of myometrial contraction, which need further experimental verification.

Additionally, we tested 10 lncRNAs to validate the reliability of RNA-seq data. By combining RNA-seq with RT-qPCR validation, LOC107985064, LINC01088, PGM5-AS1, LOC105374235, and LINC00595 were identified differently expressed between in labor and non-labor myometrium. At present, the research on the function of lncRNAs is limited. Researches on LINC01088 and PGM5-AS1 were primarily in oncology [32, 33], while lacking in myometrium. These several significantly differentially expressed lncRNAs we detected might be investigated as potential targets for uterine contraction regulation.

In this study, as part of our study design, we used a set of robust inclusion and exclusion standards to exclude variables that could interfere with or confuse our results. For example, the cases were restricted only to women who were primipara, no cesarean section or any uterine surgery before, so as to ensure that the sample tissue does not contain uterine scar tissue. That was different to the previous studies. However, there were also some limitations in this study. A limited sample size may not reveal a fully authentic expression profile of lncRNA, so larger cohorts are needed. Moreover, as many discovered lncRNAs are functionally unknown, we indirectly predicted lncRNA functions through pathway and network analyses of co-expressed protein-coding genes; the precise molecular mechanism of lncRNAs in parturition required further function-based experiments.

In conclusion, RNA-seq and bioinformatics methods were used to investigate the gene expression landscape and regulatory mechanism of lncRNAs in the myometrium during labor. A total of 112 lncRNAs were found significantly differentially expressed between the non-labor and in labor groups. Many of these lncRNAs may be involved in inflammation-related biological pathways via their regulated protein-coding target genes. Our study provided some potential lncRNA candidate targets for the related functional study of lncRNA in human labor.

## Supplementary Information

Below is the link to the electronic supplementary material.

Supplementary file1 (XLS 1108 KB)

## Data Availability

Raw and processed lncRNA sequencing data are available via the Gene Expression Omnibus (GEO) repository GSE181348.
